# Biomarkers From the Microbiome to Predict ALD Progression and Its Severity: A Comprehensive Review

**DOI:** 10.1155/mi/7244952

**Published:** 2026-07-29

**Authors:** Suraj Mishra, Harshrajsinh Solanki, Palash Mandal

**Affiliations:** ^1^ Department of Biological Sciences, P.D. Patel Institute of Applied Sciences, Charotar University of Science and Technology, Changa, Gujarat, India, charusat.ac.in

## Abstract

Alcohol use is a major global health issue, causing about 3.3 million deaths each year, or roughly 5.9% of all deaths worldwide. Alcohol‐related liver disease develops in stages: steatosis, steatohepatitis, fibrosis, cirrhosis, and hepatocellular carcinoma. If alcohol consumption is discontinued at early stages, alcohol‐related fatty liver disease can be reversed; however, continued exposure leads to progressive liver injury and increased mortality risk. Current diagnostic tools lack sufficient sensitivity and specificity to detect early‐stage disease or accurately assess disease progression. This highlights the need for reliable and mechanistically relevant biomarkers for early diagnosis and staging. This review examines alterations in gut microbiota across different stages of alcohol‐associated liver disease and evaluates gut microbiota‐associated biomarkers, including microbial metabolites, in the context of their potential diagnostic and prognostic utility. In addition, the review discusses the limitations of existing biomarkers and highlights the emerging role of microbiome‐derived signals in reflecting disease mechanisms. These findings suggest that gut microbiota‐related biomarkers may provide a promising but still evolving approach for improving early detection and understanding disease progression in alcohol‐associated liver disease.

## 1. Introduction

Excess alcohol consumption is one of the leading global health problems. It is associated with ~3.3 million deaths annually, or ~5.9% of the total number of deaths worldwide [1]. Alcohol damages most of the organs, but the liver is the most affected, as it metabolizes most of the alcohol. Regular consumption of more than 40 g of alcohol per day over time is strongly linked to the development of alcohol‐associated fatty liver disease (ALD) [2]. ALD remains a significant cause of liver‐related morbidity and mortality, representing a significant percentage of cirrhosis and liver‐related deaths worldwide [3]. The clinical spectrum of the disease includes simple steatosis to alcoholic steatohepatitis (ASH), fibrosis, cirrhosis, and hepatocellular carcinoma [4, 5]. Nevertheless, the development of the disease is very heterogeneous, unpredictable, and nonuniform. This heterogeneity indicates the interplay of a variety of factors, such as genetic predisposition, dysregulation of the immune system, metabolic condition, and environmental factors, such as diet and alcohol‐drinking patterns [6, 7]. More recently, the gut microbiota has been highly implicated as an influential player in ALD pathology, and the changes in microbial composition and functionality may affect the intestinal barrier integrity, immune signaling, and hepatic inflammation, which leads to interindividual variations in disease severity [8, 9]. These factors collectively influence both susceptibility to liver injury and the rate of disease progression, highlighting the heterogeneous nature of ALD.

Pathologically, ALD develops in a path of injury starting with steatosis and potentially progressing to steatohepatitis, fibrosis, cirrhosis, and hepatocellular carcinoma. The first phase, steatosis, is marked by the presence of triglycerides in the hepatocytes, which is located mainly in the perivenular (zone 3) area, and it is the result of metabolic imbalance caused by the chronic alcohol intake [10]. At this point, hepatocytes undergo metabolic stress and intracellular signaling pathway activation that predisposes them to injury [4]. As the consumption of alcohol continues, new pathogenic mechanisms are revealed, such as oxidative stress, disturbance of intestinal tight junctions and endotoxemia, and the activation of innate immune responses [11]. These mechanisms facilitate the infiltration of inflammatory cells into the hepatic parenchyma, signaling the transition to steatohepatitis [3]. On a histological level, this phase is characterized by hepatocellular ballooning caused by cytoskeletal damage and the development of Mallory‐Denk bodies, which is evidence of continued cellular stress [12]. If persistent, an inflammatory state can cause activation of hepatic stellate cells (HSCs), which transdifferentiate into fibrogenic myofibroblasts, producing extracellular matrix components, resulting in fibrosis of the liver. It is important to note that fibrogenesis can start quite early in vulnerable persons instead of being limited to the advanced stages [7]. Further accumulation of extracellular matrix gradually interferes with hepatic structure, which leads to cirrhosis, characterized by a nodular regeneration process and compromised liver functioning. Ultimately, cirrhosis significantly increases the risk of developing hepatocellular carcinoma, contributing to the high global mortality associated with ALD [13]. Notably, early‐stage disease, especially steatosis and steatohepatitis are potentially reversible with long‐term alcohol abstinence [14].

Even though the current understanding of the disease processes has improved, the clinical assessment of ALD remains poor. At present, ALD is most commonly diagnosed based on the drinking history of a patient, liver tests, and imaging, such as computed tomography (CT) scans, ultrasound, and magnetic resonance imaging (MRI). Nevertheless, these approaches have a restriction since more than 90% of patients have nonspecific symptoms [15]. Biochemical conventional markers lack sensitivity and specificity in early disease and cannot always be correlated with the histological extent of activity [5, 16]. Similarly, imaging techniques can detect structural abnormalities in the liver but have relatively less benefit in assessing inflammatory activity or early fibrosis. Liver biopsy is still considered the gold standard, but because of its invasive nature and risk of complications, it cannot be widely used in clinical practice [16]. Researchers should identify additional biomarkers of the various stages of the disease to overcome these challenges and enhance early diagnosis [17]. The research of these biomarkers can aid in the identification of the disease, monitoring its progress, and introducing new therapeutic approaches towards ALD.

## 2. Existing Biomarkers and Their Limitations

Biomarkers in ALD are used in a variety of ways, such as to identify liver damage, to evaluate alcohol intake, and to forecast the outcome of the disease. Historically, these biomarkers have been categorized as diagnostic, prognostic, and mechanistic, but this categorization is also being narrowed to indicate variations in clinical utility and biological significance [17, 18]. The structured framework is especially essential in ALD, where the heterogeneity of diseases and overlapping pathophysiological mechanisms make it difficult to interpret individual markers (Table 1).

**Table 1:** List of some existing biomarkers: The table lists well‐known biomarkers linked to alcohol use and liver injury, showing what they can help diagnose and where they fall short.

S. no.	Biomarker	Category	Stage	Performance	Specificity	Limitation	References
1.	AST/ALT ratio	Diagnostic	Broad spectrum (steatosis to cirrhosis)	Poor correlation with fibrosis	Low	Elevated in non‐ALD liver diseases; may be normal in advanced ALD	[5]
2.	GGT	Diagnostic	Early detection/broad spectrum	Sensitive but low predictive value	Very low	Influenced by drugs, metabolic disease, and obesity	[5]
3.	CDT	Alcohol consumption	Sustained alcohol use	Moderate sensitivity (~60%–70%)	Moderate	False positives in genetic variants and advanced disease; normalizes with abstinence	[19]
4.	PEth	Alcohol consumption	Sustained alcohol use (up to 3–4 weeks)	Superior sensitivity to CDT; unaffected by liver disease	Very high	Cannot distinguish severity of liver injury; higher assay cost	[20]
5.	EtG/EtS	Alcohol consumption	Recent alcohol exposure (<72 h)	High sensitivity; short‐term detection	High (for exposure)	Short detection window (~24–72 h); does not reflect liver injury	[21]
6.	Bilirubin	Prognostic	Advanced cirrhosis/AH	Strong predictor in severe disease	Low	Elevates late; nonspecific across liver diseases	[22]
7.	INR	Prognostic	Advanced cirrhosis/AH	Predicts mortality (used in MELD)	Low	Affected by anticoagulants and systemic factors	[22]
8.	MELD score	Prognostic	Advanced cirrhosis/AH	AUC ~0.80–0.85 (short‐term mortality)	Moderate	Limited utility in early disease; lacks mechanistic insight	[22]
9.	CK‐18 (M30/M65)	Pathophysiological	Active alcoholic hepatitis (AH)	Better correlation with histological severity than traditional enzymes	Moderate	Lacks standardized clinical thresholds; limited routine lab availability	[23]
10.	Ferritin	Pathophysiological	Inflammatory and advanced ALD	Associated with severity (no standardized cutoff)	Low	Nonspecific acute phase reactant; elevated in systemic inflammation	[24]
11.	Cytokines (TNF‐α, IL‐6)	Mechanistic	AH	Variable; reflects active inflammatory cascades	Low	High interindividual variability; lacks standardized clinical thresholds	[4, 17]
12.	Acrolein	Mechanistic	Broad spectrum (experimental)	Experimental evidence of oxidative damage	Unknown	Not validated clinically; lacks standardized high‐throughput assays	[25]

*Note:* These markers offer helpful clinical details, but they are not specific or sensitive enough to reliably stage or predict how alcohol‐related liver disease will progress.

### 2.1. Limitations of Diagnostic Biomarkers

Hepatocellular injury markers such as aspartate aminotransferase (AST), alanine aminotransferase (ALT), and gamma‐glutamyl transferase (GGT) are common in clinical practice as the first line of assessment of ALD. Another typical aspect of alcohol‐related liver damage is an increased AST to ALT ratio, which can be over 2:1, due to mitochondrial damage and pyridoxine deficiency related to chronic alcohol consumption [5]. Nevertheless, this pattern is not specific and can also be found in progressive fibrosis or cirrhosis of other etiologies. Moreover, transaminase levels are not always associated with the severity of the histology, and patients with severe ALD can have mild or even normal enzyme levels [5].

### 2.2. Limitations of Alcohol Consumption Biomarkers

Carbohydrate‐deficient transferrin (CDT), ethyl glucuronide (EtG), and ethyl sulfate (EtS) are examples of biomarkers of alcohol consumption that offer objective data on alcohol consumption. CDT indicates long‐term high alcohol intake with the glycosylation of transferrin, whereas EtG and EtS are direct ethanol metabolites that can be measured within a short time after alcohol intake [21]. As a result, their use in clinical management is primarily for marking abstinence or relapse, and they are not useful for assessment of disease activity, severity, or prognosis [19].

### 2.3. Limitations of Prognostic Models

The severity of ALD is assessed mainly based on composite clinical scores, as opposed to single biomarkers. Short‐term mortality of patients with advanced ALD and cirrhosis has been predicted by the model for end‐stage liver disease (MELD), which includes serum bilirubin, creatinine, and international normalized ratio (INR) [22]. In a similar fashion, the Maddrey discriminant function is used to determine patients with severe alcoholic hepatitis (AH) who can be treated with corticosteroids, and the Lille score is used after treatment to evaluate early response and decide whether to continue or discontinue corticosteroids [24, 26]. Although these models have clinical use, they are essentially founded on parameters that indicate hepatic dysfunction, as opposed to disease pathophysiology. Consequently, they have limited utility in detecting patients who may develop the disease at earlier stages of ALD. Moreover, their performance may vary across patient populations and clinical settings, limiting their generalizability [27].

### 2.4. Limitations of Pathophysiological and Mechanistical Biomarkers

Pathophysiological biomarkers can give information about the mechanisms of alcohol‐induced liver injury, but they are not regularly implemented in practice. Serum ferritin is often increased in ALD and has been linked to the severity of the disease, as it indicates hepatic iron overload and systemic inflammation [28]. Tumor necrosis factor‐alpha (TNF‐a) and interleukin‐6 (IL‐6) are some of the inflammatory cytokines that are important in the pathogenesis of AH and are associated with inflammatory activity, but their clinical use is limited by variability and the absence of standardized thresholds [7]. Markers of oxidative stress, including acrolein, a reactive aldehyde produced during ethanol metabolism and lipid peroxidation, also demonstrate that the current biomarkers do not capture upstream processes driving ALD progression, thus necessitating the investigation of microbiome‐derived biomarkers in the gut–liver axis [29].

## 3. Gut–Liver Axis and Gut Dysbiosis

The gut–liver axis represents a bidirectional communication system between the intestine and the liver, primarily mediated through the portal circulation. Nutrients, microbial metabolites, and other luminal products absorbed across the intestinal barrier are transported directly to the liver, where they influence metabolic and immune responses [30]. The intestinal barrier at a physiological level selectively allows transportation of nutrients but limits translocation of microorganisms and microbial‐associated molecular patterns (MAMPs), sustaining homeostasis of the immune system [31]. Structurally, this barrier consists of a mucus layer, epithelial cells, and immune components. Intestinal epithelial cells are linked by tight junctions, which consist of a range of proteins, including the claudins, occludin, and members of the family of zonula occludens (ZO‐1 and ZO‐2). Together, they regulate the permeability of the paracellular space and are responsible for barrier integrity [32, 33]. The disruption of this barrier represents a key early event in the pathogenesis of ALD.

**Figure 1:**
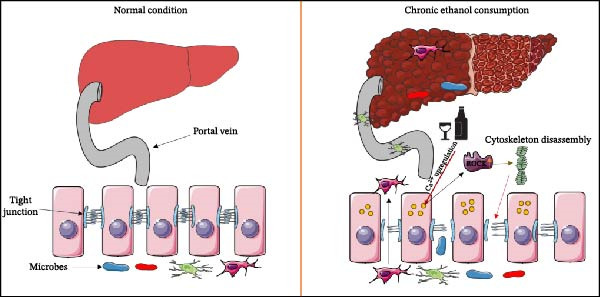
Gut–liver axis in alcoholic liver disease. In the left panel, the gut–liver axis is healthy because tight junctions between epithelial cells maintain a strong barrier and prevent microbes from passing through. In the right panel, alcohol disrupts this balance by affecting tight junctions through these pathways, making the intestine more permeable. This allows microbial products to enter the portal vein, triggering immune cells in the liver and causing inflammation. Over time, this process can lead to alcohol‐associated liver disease.

Persistent exposure to ethanol disrupts the normal functioning of the intestinal barrier both directly and indirectly. Ethanol and its major metabolite acetaldehyde interfere with the tight junction integrity by altering the protein structure and localization. Moreover, ethanol causes endoplasmic reticulum (ER) stress and elevates intracellular calcium, triggering the RhoA/Rho‐associated protein kinase (ROCK) signaling pathway (Figure 1), resulting in cytoskeletal rearrangement and disassembly of tight junctions [34]. All these alterations result in a more permeable intestine, which allows microbes and their products like lipopolysaccharide (LPS) to be translocated to the portal circulation. At the same time, alcohol intake also influences the composition and function of the gut microbiota, leading to dysbiosis. Several studies have indicated a decrease in the microbial diversity and changes in the microbial composition, such as an increase in the abundance of Proteobacteria and changes in the Firmicutes and Bacteroidetes phyla [35]. However, these compositional changes are not entirely consistent across studies and are influenced by factors such as diet, disease stage, and host variability. Notably, these changes are not only taxonomic but also functional since they are accompanied by a decrease in the production of favorable microbial metabolites, especially short‐chain fatty acids (SCFAs), and an increase in endotoxins.

The combined effects of barrier dysfunction and dysbiosis result in enhanced translocation of microbial‐derived products to the liver, where they activate innate immune pathways, including Toll‐like receptor signaling, leading to inflammation and disease progression [36]. From a biomarker perspective, this process generates measurable indicators such as circulating LPS, soluble CD14, microbial metabolites, and shifts in microbial composition, which reflect underlying gut–liver axis dysfunction. Taken together, disruption of the gut–liver axis provides a mechanistic link between alcohol exposure, intestinal permeability, microbial dysbiosis, and hepatic inflammation. Importantly, these interconnected changes form the basis for identifying microbiota‐derived and gut‐associated biomarkers across different stages of ALD [37–39]. Given this mechanistic framework, understanding how gut microbiota composition changes across different stages of ALD is critical for identifying stage‐specific biomarkers and assessing the disease progression.

Microbial changes in early ALD, with alcohol‐associated steatosis, are relatively subtle and heterogeneous. Human studies of chronic alcohol consumption and early disease have reported reductions in beneficial taxa such as Faecalibacterium and members of Ruminococcaceae, alongside enrichment of Proteobacteria and other Gram‐negative bacteria [40, 41]. These findings are further supported by experimental models, which show a decreased abundance of Firmicutes and Lactobacillus after ethanol exposure. These taxa are mainly responsible for the production of SCFAs, especially butyrate, which enhances intestinal barrier integrity [42, 43]. Nevertheless, in human groups, these changes are mainly associative and demonstrate significant interindividual differences. Moreover, numerous studies lack a clear stratification of disease stage, which restricts the application of early patterns of dysbiosis as effective biomarkers of steatosis.

Stage‐specific signatures are inconsistent, but dysbiosis intensifies as ALD advances towards steatohepatitis. It has been reported to increase the abundance of potentially pathogenic taxa, including Enterococcus, Streptococcus, and Enterobacteriaceae, and further reduce the abundance of SCFA‐producing bacteria (Table 2) [35, 41]. These compositional changes have been linked with increased intestinal permeability and circulating LPS, exacerbating activation of hepatic inflammatory pathways, including TLR4/NF‐B signaling [48, 49]. Although mechanistic models provide evidence of a role of gut‐derived endotoxemia in liver inflammation, human evidence has shown more correlations between microbial changes and inflammatory markers than causation. Additionally, beta diversity comparisons tend to identify similar microbial communities between steatosis and steatohepatitis, suggesting that microbiota composition alone lacks discriminatory ability to differentiate between intermediate disease stages [50].

**Table 2:** Alterations in gut microbiota among patients with alcoholic liver disease: A research investigation.

Study	Model	Methodology	Key microbial changes	Interpretation	Confounders
Alcohol associated steatosis					
Patients (*n* = 24, mean age 64.8), Alcohol overconsumption history (>10 years) [40]	Human	16S rRNA sequencing	↑Proteobacteria (*Sutterella*, *Clostridium*), *↓ Faecalibacterium*	↓SCFA producing taxa – early epithelial barrier impairment and reduced butyrate availability	Diet, alcohol intake
Intragastric administration of ethanol in C57/B6 mice for 3 weeks [44]	Mice	16S rRNA sequencing	↑Bacteroidetes, Verrucomicrobia; ↓Firmicutes, *Lactobacillus*	Reduced butyrate production – impaired tight junction integrity and increased permeability	Controlled conditions
21 patients samples with ALD‐steatosis (longer history of alcohol consumption) [41]	Human	16S rRNA sequencing	↑ Enterobacteriaceae; ↓ Ruminococcaceae	Enrichment of LPS‐producing bacteria‐low grade endotoxemia and immune activation	Antibiotics not controlled
C57BL/6 (*n* = 7) modified Lieber‐Decarli liquid diet was given for 8 weeks to induce ALD. [43]	Mice	16S rRNA sequencing	*↑ Enterococcus*, *Streptococcus*	Expansion of pathobionts‐ increased inflammatory signaling and epithelial disruption	Controlled model
Alcoholic cirrhosis					
244 human subjects (43 alcoholic cirrhosis) between age 55 and 58 years [45]	Human	16s rRna sequencing	*↑Enterobacteriaceae and Veillonella*	↑Endotoxin‐producing taxa –systemic inflammation and HE risk	Medications, hospitalization
170 patients (24 alcoholic cirrhosis) with history of alcohol consumption >5 year and mean age between 55 and 58 years [46]	Human	16 s rRna sequencing	*↑Enterococcus, Sphingomonas, Proteobacteria*	Increased pathogenic taxa linked to immune activation	Comorbidities
36 liver cirrhosis patients (12 alcohol‐related) with mean age of 49 ± 11 [42]	Human	16 s rRna sequencing	*↓Lachnospiraceae, Ruminococcaceae, Prevotellaceae*	Loss of SCFA producers –barrier dysfunction and endotoxemia	Small cohort
99 patients (27 alcoholic cirrhosis) with age 20 to 60 years old [47]	Human	16 s rRna sequencing	*↑Bacteroides, Streptococcus;* *↓ Prevotella*	Shift toward pro‐inflammatory microbiota and reduced metabolic diversity	Diet, geography

In contrast, advanced ALD, particularly cirrhosis, is associated with more consistent and reproducible dysbiosis signatures. Various studies document significant losses of microbial diversity, as well as the loss of beneficial commensals, including Lachnospiraceae, Ruminococcaceae, and Prevotellaceae, with an increase of pathogenic taxa like Enterobacteriaceae, Veillonella, and Streptococcus [42, 45, 47]. These alterations are accompanied by functional consequences, including reduced SCFA production, increased endotoxin burden, and systemic inflammation. Notably, microbiota profiles in cirrhosis have been linked to complications and severity of the disease. As an illustration, the enriched state of pathogenic taxa and the decreased diversity have been associated with the elevated model of end‐stage liver disease (MELD) scores and the risk of hepatic encephalopathy (HE) and hospitalization [45]. Nevertheless, these associations are not consistently found in all the studies, and microbiome‐based measures have not always shown independent predictive value of other existing clinical scoring systems.

From a biomarker perspective, these findings suggest that gut dysbiosis signatures are more robust indicators of disease severity than of precise stage differentiation, particularly in early and intermediate ALD. Besides, the vast majority of human investigations are cross‐sectional, with fairly small cohorts, and are confounded by diet, exposure to antibiotics, use of proton pump inhibitors, and continued alcohol intake. Differences in sequencing methods and analysis pipelines make it hard to compare results across different studies. Microbiota‐based biomarkers alone are not likely to be useful in clinical settings. Their value comes from combining them with functional and host‐derived markers like LPS, soluble CD14, CRP, and cytokines. This combined approach better captures the complexity of ALD and may predict outcomes more accurately than using single microbial features.

## 4. Gut‐Derived Microbial Metabolites in Alcohol‐Associated Liver Disease

Chronic alcohol exposure disrupts multiple interconnected pathways within the gut–liver axis, including intestinal barrier integrity, microbial composition, bile acid metabolism, and host immune recognition of microbial products. These alterations lead to changes in the production, absorption, and systemic effects of gut‐derived microbial metabolites, which play a critical role in the progression of ALD [7]. Among these metabolites, SCFAs are particularly important due to their central role in maintaining intestinal homeostasis and modulating hepatic metabolism.

### 4.1. SCFAs

SCFAs are produced through the microbial fermentation of dietary fiber in the colon, as these substrates escape digestion in the upper gastrointestinal tract [51]. Acetate, butyrate, and propionate are the three primary SCFAs; in the intestines, their combined concentration can exceed 100 mM. Bacteroidetes are the most important contributors to acetate and propionate synthesis, and Firmicutes contribute to the production of butyrate [52]. The large intestine and cecum absorb most of the SCFAs, with the remainder being excreted in feces [53]. SCFAs act as an energy source for colonocytes and help maintain a tight barrier by supporting tight junction proteins and mucin secretion in the gut [54].

SCFAs such as propionic acid and butyric acid interact with GPR43 and GPR41 on immune cells and IECs, inhibiting lipid MAPK phosphorylation and NF‐κB activity. This leads to a decrease in inflammatory factors like iNOS, TNF‐α, MCP‐1, and IL‐6, reducing immune cell recruitment and producing anti‐inflammatory effects [55]. Additionally, butyrate activates GPCR109A in antigen‐presenting cells and downregulates the gene expression of proinflammatory cytokines and chemokines [56]. Overall, butyrate supports an anti‐inflammatory environment and maintains intestinal balance by reinforcing tight junctions, increasing mucin production in goblet cells, boosting antimicrobial peptides in Paneth cells, and promoting the differentiation of anti‐inflammatory regulatory T‐cells (Tregs) [34]. SCFAs travel to the liver through the portal vein, where they act as substrates for lipid or glucose metabolism. For example, acetate helps in lipogenesis, while propionate suppresses cholesterol and lipid synthesis, and butyrate can activate AMPK signaling and reduce fat accumulation in hepatocytes [57–59]. Alcohol intake alters the gut microbiota, reducing SCFAs producing anaerobic bacteria such *as A. muciniphila*, *Bacteroides vulgatus*, *Bacteroides thetaiotaomicron*, and *Coprococcus catus* [60]. In ALD, reduced beneficial microbes such as *Faecalibacterium prausnitzii* cause lower butyrate and propionate levels, and this weakens the gut barrier and increases intestinal permeability [61]. This impairment of intestinal barrier integrity enhances permeability and enables translocation of bacterial endotoxins like LPS to exacerbate hepatic inflammation.

Interestingly, some studies have reported no significant difference in SCFA concentrations during the early phases of ALD, which have been attributed to possible compensatory absorption of SCFAs by the host or minimal dysbiosis at this stage, but as the disease progresses to the steatohepatitis stage, more gut dysbiosis leads to reduced SCFA production and increased hepatic inflammation and endotoxemia. In a study, Cao et al. [62] reported that in alcoholic cirrhosis, total fecal SCFAs average 5.31 mg/g (vs. 10.2 mg/g in controls), with acetate at 3.14 mg/g (vs. 5.87 mg/g), propionate at 1.11 mg/g (vs. 1.77 mg/g), and butyrate at 0.68 mg/g (vs. 1.69 mg/g), all significantly lower (*p* <0.001). These results confirm the correlation between progressive dysbiosis, decreased SCFA generation, and hepatic inflammation in ALD. All these observations suggest that SCFAs have a dual action in ALD, as they are both regulators of the intestinal barrier function and regulators of hepatic metabolism. Their loss in progressive disease underscores their possible use as mechanistic biomarkers and therapeutic targets of the gut–liver axis. Nevertheless, data on SCFAs are obtained, primarily, due to the preclinical research. To confirm the SCFA level at all stages of ALD, the clinical research is required.

### 4.2. BAs

Primary bile acids (BAs) like chenodeoxycholic acid (CDCA) and cholic acid (CA) are amphipathic molecules produced from liver‐secreted cholesterol and modified by gut bacteria [63]. The colon’s resident microbiota converts the remaining primary BAs into secondary bile acids, namely, deoxycholic acid (DCA) and lithocholic acid (LCA) [64]. Primary bile acids maintain intestinal integrity, glucose homeostasis, and lipid metabolism by signaling through the farnesoid X receptor (FXR) and Takeda G protein‐coupled receptor 5 (TGR5) [65]. FXR activation exerts several anti‐inflammatory effects; for example, it attenuates colitis by suppressing proinflammatory genes, which lowers the expression of proinflammatory cytokines [66, 67]. The G protein bile acid receptor (TGR5) is mostly activated by secondary bile acids such as LCA and DCA, and its activation in intestinal monocytes and macrophages triggers them to produce the IL‐10 cytokine, exhibiting anti‐inflammatory activity [68, 69].

High concentrations of bile salts can quickly break down membrane lipids and cause integral membrane proteins to dissociate. Hydrophobic BAs at high concentrations are known to have antimicrobial activity by rupturing the bacterial membrane, and secondary BAs usually have a higher toxicity to bacteria than primary BAs [64].

People with ALD often show higher total bile acids (TBAs) in the blood, but the liver makes and secretes less of the normal bile acid pool, which also alters how bile acids interact with gut microbes and gut barrier function [55]. According to recent reviews, lower luminal bile acid concentrations reduce FXR activation in intestinal cells, weakening the enteric epithelial lining and allowing pathogens to proliferate [65]. In case of alcoholic steatosis, TBAs in the serum increases which inhibit mitochondrial β‐oxidation by downregulating the expression of PPARα, SIRT1, and CPT1(carnitine palmitoyltransferase 1) genes and promote lipid uptake via the CD36 pathway [70]. Taurochenodeoxycholate, glycocholate, taurocholate, tauroursodeoxycholate, and taurolithocholate 3‐sulfate were increased significantly in alcohol‐associated hepatitis [71]. Researchers also found that total and conjugated BAs are markedly elevated in AH patients, and de novo synthesis is inhibited due to a drop in CYP7A1 gene expression and C4 serum levels [30, 72]. In the case of cirrhosis, alcohol consumption is linked to a marked increase in secondary BA production [73].

From a biomarker standpoint, alterations in circulating and fecal bile acid profiles suggest potential utility in reflecting the disease stage and metabolic dysregulation in ALD. However, their clinical application remains limited due to interindividual variability, influence of diet and microbiota composition, and lack of standardized thresholds. In addition, bile acid alterations overlap with other liver diseases, reducing their specificity as standalone biomarkers.

### 4.3. Ammonia

Ammonia is produced in the colon by a number of gut microbial species, either by proteolysis or the conversion of urea by bacterial urease. Bacteria produce urease, an enzyme protein that helps convert urea into carbamate and ammonia. Since there is no known urease gene in mammals, urease‐assisted urea breakdown in the colon depends solely on the gut bacteria [74]. Several bacteria, like *Klebsiella pneumonia*, *Staphylococcus aureus*, and *Helicobacter pylori* are abundant in cirrhosis and contribute to excess ammonia by urease activity [74, 75]. In addition to urease, a variety of bacterial species in the colon, like *Staphylococcaceae*, *Enterobacteriaceae*, and *Lactobacillaceae*, produce ammonia by proteolytic activity [76, 77].

The liver effectively detoxifies ammonia by converting it into glutamine via glutamine synthetase (GS) or into urea through the urea cycle enzymes [78]. As liver disease progresses, hepatic urea synthesis is impaired, reducing ammonia clearance capacity. In addition, extrahepatic sources contribute to ammonia accumulation; for example, increased intestinal glutaminase activity and renal glutamine catabolism further elevate circulating ammonia levels, leading to hyperammonemia [79]. Ammonia can cross the blood–brain barrier, causing neutrophil dysfunction and ROS generation, which can induce oxidative stress and neuroinflammation [80]. A range of neuropsychiatric and neurological symptoms, including impaired memory, brain edema, seizures, ataxia, and coma, are caused by elevated ammonia concentrations in the brain due to hyperammonemia [81]. HE is primarily caused by hyperammonemia, which is brought on by poor hepatic clearance and portosystemic shunting in cirrhosis [82]. The primary cells impacted during HE in cirrhosis patients are astrocytes and microglial cells [83]. Elevated ammonia concentrations depict immunosuppressive effects by reducing natural killer (NK) cell cytotoxicity, suppressing T cell growth and function, and compromising immunological surveillance [84]. Besides its neuro‐effect, it has also been suggested in hepatic injury since ammonia can induce the proliferation and functioning of HSCs, leading to fibrogenesis [85]. Cirrhosis, which is a significant risk factor for the development of HCC, can result from progressive fibrosis. Furthermore, ammonia directly causes hepatotoxicity by triggering inflammatory reactions and oxidative stress, which promotes tumor cell invasion, survival, and metastasis [85, 86].

Ammonia is widely used in the clinical assessment of HE; however, its utility is limited by variability in correlation with the clinical severity and influence of extrahepatic factors. Blood ammonia levels do not always correlate with the extent of neurological damage and thus may not serve as a standalone marker for disease stages or prognosis.

### 4.4. Amino Acids/Tryptophan Products

Amino acids act as intermediate metabolites that influence the production of lipids, glutathione, nucleotides, glucosamine, and polyamines. They also play a role in cell growth and the movement of carbon in the tricarboxylic acid cycle [87]. The liver is central to amino acid metabolism, protein synthesis and degradation, and several detoxification processes, particularly those involving end‐products of intestinal metabolism such as ammonia [88]. Branched‐chain amino acids (BCAAs: leucine, valine, and isoleucine) are a class of important amino acids that have aliphatic branched side chains. BCAAs support the energy balance, including gluconeogenesis and lipid metabolism, in addition to serving as a necessary substrate for protein synthesis [89, 90]. This implies that liver problems may be caused by the disturbance of the homeostasis of BCAAs.

Host cells convert dietary tryptophan into serotonin, melatonin, and kynurenine, whereas gut bacteria convert it into indoles and their derivatives, such as indole‐3‐propionic acid (IPA) and indole‐3‐acetic acid (IAA) [63, 91, 92]. These indoles serve as ligands for the intestinal aryl hydrocarbon receptor (AHR), maintaining the gut barrier integrity. Chronic alcohol consumption induces gut dysbiosis, depleting production of these indoles, which causes reduced AHR signaling, leading to increased intestinal permeability, thus allowing bacterial translocation into the liver, causing inflammation and steatosis [93]. In AH, the enzyme IDO1 (Indoleamine 2,3‐dioxygenase 1) is significantly upregulated by interferon γ, TNFα, and other cytokines—inflammatory cytokines [94]. This diverts tryptophan from protein synthesis to kynurenine, which acts as an immunosuppressant by promoting T‐cell exhaustion and Treg differentiation [95]. Despite the suggested use of the kynurenine/tryptophan ratio as a possible prognosis biomarker, the existing evidence is still limited, and the correlation between the ratio and other established clinical predictors, such as the MELD score or short‐term mortality in AH, has not been proven to be consistently valid in various cohorts [96]. Therefore, its clinical applicability remains preliminary and requires further validation.

As the disease progresses to cirrhosis, kynurenine is further metabolized to quinolinic acid (QA), which crosses the blood–brain barrier and acts as a neurotoxin contributing to HE [97]. It has also been found that serotonin levels significantly drop in a cirrhotic liver, which accelerates fibrosis and impairs liver regeneration [98].

Tryptophan metabolites such as kynurenine and indole derivatives have shown promise as potential biomarkers for immune dysregulation and gut–liver interactions in ALD. However, the application of such metabolites to clinical practice still remains challenging due to biological variability of metabolites, impact of microbiota on metabolite levels, and absence of established diagnostic thresholds.

## 5. Gut–Microbiota Associated Biomarkers

Chronic ethanol consumption causes intestinal dysbiosis, leading to increased intestinal permeability. Increased permeability enhances the susceptibility of microbial antigens to immune response. Hence, gut dysbiosis dysregulates immune homeostasis in the intestine [99].

During the early stage of alcohol ALD in steatosis, gut microbial diversity is affected, and beneficial microbes are reduced. In steatosis, not only is the gut microbiota affected, but intestinal permeability is also altered as tight junction proteins are disturbed. As a result, mild endotoxemia can be seen in this stage, characterized by elevated levels of LPS, indicating dysregulation of tight junctions and affected enterocytes. Moreover, activation of Kupffer cells through the Toll‐like receptor‐4 (TLR4) pathway is observed with lower upregulation of cytokines. Increased LPS also leads to an enhanced soluble CD14 level, denoting an active immune response. Additionally, biomarkers like zonulin and intestinal fatty acid‐binding proteins are upregulated in the steatosis stage [49, 100, 101]. These markers can indicate early barrier dysfunction, but their clinical usefulness is restricted as they are not very specific and vary in different patients.

**Figure 2:**
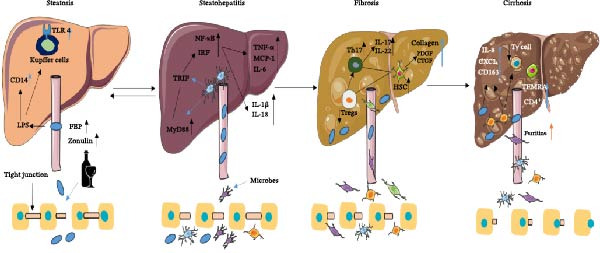
Immune responses across the stages of alcoholic liver disease. In the steatosis stage, alcohol seems to make the intestine more permeable, which allows LPS to move into the portal blood and activate Kupffer cells through TLR4/CD14. When this shifts toward steatohepatitis, several inflammatory pathways (for example, NF‐κB, IRF, TRIF, and MyD88) start to play a larger role, and cytokines such as TNF‐α, IL‐6, and MCP‐1 increase, bringing more immune cells into the liver. In fibrosis, there is usually an imbalance between Th17 and Treg cells and activation of hepatic stellate cells, which results in collagen deposition. Cirrhosis appears as the more advanced stage, where chemokines (like IL‐8 and CXCL types), soluble CD163, and changes in T cell populations are more noticeable, along with stronger dysbiosis.

While in the steatohepatitis stage, severe endotoxemia is observed, marked by a higher concentration of LPS and CD14 found in steatosis. And ultimately, because of excessive endotoxemia, severe activation of pathogen‐associated molecular patterns (PAMPs), specifically TLR‐4 and TLR‐9, are upregulated and further lead to activation of both MyD88‐dependent and TRIF‐dependent signaling cascades, causing activation of NF‐κB and IRF transcription factors [102]. Activation of these transcription factors triggers cascades of proinflammatory cytokines, including TNF‐α, IL‐6, MCP‐1, IL‐1β, and IL‐18 (Figure 2). Another key inflammatory pathway that is upregulated in steatohepatitis is the NLRP3 inflammasome, which is triggered by gut‐derived endotoxins, ethanol metabolites, and mitochondrial stress signals, leading to caspase‐1–mediated activation of IL‐1β and IL‐18 [103, 104]. Activation of these cytokines further amplifies inflammatory signaling. Moreover, macrophage polarization toward proinflammatory activity is often elevated, as reflected by increased soluble CD163 levels [48]. These inflammatory markers are associated with disease activity but are not unique to ALD and are also found to be increased in other inflammatory and hepatic diseases.

Disease progression from steatohepatitis to fibrosis results in activation of HSCs. Activation of HSCs is associated with upregulation of transforming growth factor‐β, platelet‐derived growth factor, and connective tissue growth factor [105, 106]. HSCs produce type I and type III collagen, fibronectin, and laminin, leading to distorted liver architecture. Additionally, apart from an altered innate immune response, a distorted adaptive response is indicated by elevated levels of IL‐17 and IL‐22, driven by increased differentiation of Th17 cells and suppression of Tregs characterized by reduced FOXP3 expression [107, 108]. Such immune changes are indicative of progressive disease, but they are not sufficiently specific to be effective biomarkers of fibrosis on their own.

In the advanced cirrhosis stage of alcohol‐associated liver disease, dysregulation is observed among T cell populations with significant reduction of Tγ cells and upregulation of T effector memory cells re‐expressing CD45RA (TEMRA CD4+ cells) [109]. Additionally, increased polarization of Th2 cells impairs antigen‐specific responses. Moreover, chronic exposure to microbial products leads to elevated levels of IL‐8, CXCL chemokines, soluble CD163, and ferritin compared to earlier disease stages [110, 111]. While these markers may reflect advanced immune dysregulation, they are influenced by multiple systemic factors and therefore have limited diagnostic specificity.

Other immune biomarkers such as TNF‐α, IL‐6, IL‐1β, soluble CD163, and various chemokines, although elevated across different stages of ALD, are not specific and are also observed in other liver diseases, including viral hepatitis and NAFLD [112, 113]. Although upstream signaling pathways including TLR‐4 stimulation and gut‐derived endotoxemia are central in the pathogenesis of ALD, their quantification is not adequate in clinical resolution of disease stages. Even though circulating inflammatory cytokines are not specific, a combination of these measurements with gut microbiota profiles and microbial metabolites can provide a better understanding of the severity and progression of the disease but not as independent biomarkers [9, 17].

Although several microbiota‐derived products and inflammatory mediators were proposed as diagnostic markers in ALD, their translational potential is hampered by the absence of validation and comparison to proven markers of prognosis [114]. It is important to note that none of these markers are yet demonstrated to have better predictive ability compared to normal models (MELD, Child‐Pugh) or commonly used in clinical practice. Therefore, most microbiota‐based interventions can be viewed as investigational, as opposed to clinical.

## 6. Conclusion

In summary, alcohol‐associated liver disease impacts not only hepatocytes but the entire gut–liver axis. Chronic alcohol consumption disrupts the gut microbiome, compromises intestinal integrity, and allows microbial products to translocate from the gut to the liver, where they stimulate immune responses. This results in inflammation and, with continued alcohol exposure, can progress to fibrosis and ultimately cirrhosis. These complex interactions have led researchers to explore biomarkers beyond traditional liver enzymes. Accordingly, increasing attention has been directed toward gut‐derived factors, including microbial composition, metabolites such as SCFAs, bile acids, ammonia, and tryptophan pathway products, as well as immune markers like LPS, sCD14, sCD163, and various cytokines, to improve early detection and disease stratification. Nevertheless, several of these biomarkers remain at an early stage of clinical translation, as available human studies are often limited in size, and factors such as diet, medications, adiposity, and comorbid conditions can significantly influence microbiome‐related measurements. In addition, many of these markers lack disease specificity and standardized thresholds, which limits their immediate applicability in clinical practice.

In the future, integrative approaches combining multiple biomarkers rather than relying on a single marker may facilitate noninvasive assessment of disease progression and reduce reliance on liver biopsy. Consequently, the microbiome and its derived metabolites represent a promising but still evolving area for noninvasive diagnosis and prognostication in alcohol‐associated liver disease. However, large‐scale validation studies and standardization of analytical methods are required before these biomarkers can be routinely implemented in clinical settings.

## Author Contributions

**Suraj Mishra:** writing – original draft, conceptualization, literature review, editing. **Harshrajsinh Solanki**: writing – original draft, conceptualization, literature review, editing. **Palash Mandal:** verified and discussed the concepts, editing, supervision.

## Funding

No funding was received for conducting the review work.

## Conflicts of Interest

The authors declare no conflicts of interest.

## Data Availability

All data presented in this review are from previously published studies and are cited accordingly; no new data were generated.
